# Parliament and the Professions

**Published:** 1920-07-31

**Authors:** 


### Parliament and the Professions.

### Indian Medical Service.

The Secretary of State for India has issued a Press communiqué, dated July 5, which contains the revised rates of pay for the Indian Medical Service, and a copy of a letter from the Indian Office to the British Medical Association, dealing with the pay, allowances, status, etc., of the Director-General Surgeons-General.

### Income Tax and Charities.

Sir H. Nield asked the Chancellor of the Exchequer if he will consider the desirability of inserting a clause in the Finance Bill which will more clearly define what is a charity within the meaning of the existing Finance Acts, so that institutions not run for profit, yet deriving a portion of their incomes from relatives and friends of the inmates, may be exempted from payment of income tax under Schedule A on freehold and leasehold properties purchased by them out of funds subscribed by the public or from testamentary bequests, and from payment of tax under Schedule D on dividends from investments similarly acquired?

Mr. Chamberlain replied that the legal definition of the term "charity" was considered by the Royal Commission on the Income Tax, who expressed the opinion that, for the purposes of income tax, a "charity" should be specially defined by Parliament. This and other suggestions made by the Royal Commission (in Section XIV. of Part III. of the Report) in regard to the income-tax exemptions that are granted to charities will be considered when it is possible to deal with the Royal Commission's recommendations which are still outstanding.

